# Pathogenic *SMAD6* variants in patients with idiopathic and complex congenital heart disease associated pulmonary arterial hypertension

**DOI:** 10.1038/s41525-025-00484-6

**Published:** 2025-03-25

**Authors:** Sofia Karl, Ekkehard Grünig, Memoona Shaukat, Matthias Held, Christian Apitz, Fabian von Scheidt, Ralf Geiger, Michael Halank, Karen M. Olsson, Marius M. Hoeper, Jan C. Kamp, Gabor Kovacs, Horst Olschewski, Hans-Jürgen Seyfarth, Katrin Milger, Ralf Ewert, Hans Klose, Benjamin Egenlauf, Panagiota Xanthouli, Katrin Hinderhofer, Christina A. Eichstaedt

**Affiliations:** 1 Center for Pulmonary Hypertension, Thoraxklinik Heidelberg gGmbH at Heidelberg University Hospital and Translational Lung Research Center Heidelberg (TLRC), German Center for Lung Research (DZL), Heidelberg, Germany; 2 Laboratory for Molecular Genetic Diagnostics, Institute of Human Genetics, Heidelberg University, Heidelberg, Germany; 3 Department of Pulmonary Medicine, KWM Missio Clinic, Würzburg, Germany; 4 Department for Pediatric Cardiology, University Hospital Ulm, Ulm, Germany; 5 Department of Congenital Heart Disease and Pediatric Cardiology, German Heart Center Munich, Munich, Germany; 6 Pediatrics III (Cardiopulmonary Unit), Department of Child and Adolescent Health, Medical University Innsbruck, Innsbruck, Austria; 7 Devision of Pulmonology, Medical Department I, University Hospital Carl Gustav Carus of TU Dresden, Dresden, Germany; 8 Department of Respiratory Medicine and Infectious Diseases, Hannover Medical School, Hannover and Biomedical Research in Endstage and Obstructive Lung Disease Hanover (BREATH), German Center for Lung Research (DZL), Hannover, Germany; 9 Division of Pulmonology, Department of Internal Medicine, Medical University of Graz and Ludwig Boltzmann Institute for Lung Vascular Research, Graz, Austria; 10 Department of Pneumology, Medical Clinic II, University Hospital of Leipzig, Leipzig, Germany; 11 Department of Internal Medicine V, Ludwig-Maximilian University of Munich; Asklepios Clinic Gauting, Comprehensive Pneumology Centre Munich (CPC), German Center for Lung Research (DZL), Munich, Germany; 12 Department of Internal Medicine B-Cardiology, Intensive Care, Pulmonary Medicine and Infectious Diseases, University of Greifswald, Greifswald, Germany; 13 Department of Pneumology, Department of Medicine II, University Medical Center Hamburg-Eppendorf, Hamburg, Germany; 14 Department of Internal Medicine V: Hematology, Oncology and Rheumatology, University Hospital Heidelberg, Heidelberg, Germany

**Keywords:** Genetic testing, Medical genetics

## Abstract

In patients with complex congenital heart disease (CHD) pathogenic *SMAD6* variants have been described previously. The aim of this study was to analyze if pathogenic *SMAD6* variants also occur in patients with CHD associated with pulmonary arterial hypertension (CHD-APAH) or idiopathic PAH. A PAH gene panel with up to 64 genes including *SMAD6* was used to sequence 311 patients with idiopathic PAH (IPAH) and 32 with CHD-APAH. In 4 of 32 (12.5%) CHD-APAH and in 2 out of 311 (0.64%) IPAH patients we identified likely pathogenic or rare *SMAD6* missense variants. All CHD-APAH patients with a rare *SMAD6* variant had complex CHD. One patient had bi-allelic *SMAD6* variants, combined pulmonary valve defect and supravalvular aortic stenosis, craniosynostosis and radioulnar synostosis. This is the first description of potentially disease-causing *SMAD6* variants in patients with IPAH and complex CHD-APAH. Further studies are needed to assess pathogenesis and prevalence of pathogenic *SMAD6* variants in PAH.

## Introduction

Pulmonary arterial hypertension (PAH) occurs in about 4% of patients with congenital heart disease (CHD)^1–3^, particularly in those with shunt defects^4^. Patients may develop PAH years after surgical repair and are then diagnosed as PAH associated with CHD (CHD-APAH). PAH leads to an increased morbidity and mortality in CHD patients compared to those without PAH^5^.

In most patients with PAH a genetic background can be assumed^6,7^. The majority of the established PAH genes is part of the bone morphogenetic protein receptor 2 (BMPR2) signaling pathway. The *BMPR2* gene itself accounts for around 65% of pathogenic variants in PAH patients^8^. Additionally, downstream genes of BMPR2 including the SMAD proteins such as SMAD8, encoded by *SMAD9*, can also play a causal role in PAH development^9^. SMAD6 is one of two inhibitory SMAD proteins of the transforming growth factor beta (TGF-β) superfamily and has so far not been clearly associated with PAH development. However, pathogenic *SMAD6* variants have been identified in more than 20 CHD patients without PAH^10^. The most frequently described anomalies in patients with pathogenic *SMAD6* variants include left ventricular outflow tract defects such as coarctation of the aorta, bicuspid aortic valves and conotruncal defects such as tetralogy of Fallot or transposition of the great arteries^11–13^. Additionally, craniosynostosis and radioulnar synostosis have been described in patients with pathogenic *SMAD6* variants^14,15^.

In about 4–8% of CHD-APAH patients, PAH-related, pathogenic variants have been identified, particularly in the two transcription factors T-box factor 4 (*TBX4*) and the SRY box transcription factor 17 (*SOX17*)^7,8,16^. Rare deleterious *SOX17* variants have been reported in up to 3.2% of CHD-APAH cases and *TBX4* variants in 2.6% of CHD-APAH patients^16^. Almost all previously described CHD-APAH patients with pathogenic *SOX17* or *TBX4* variants had simple, isolated heart defects with an unreported surgical repair status including atrial septal defects (ASD), ventricular septal defects (VSD) and/or patent ductus arteriosus (PDA)^8,16–18^. Only a single *SOX17* patient presented with ASD, VSD, atrioventricular canal defect, mitral cleft, and situs inversus^16^.

The previous findings and publications still leave many CHD-APAH patients without a known genetic cause for their disease, including those CHD-APAH patients with an abnormal development of the pulmonary vasculature. The scarcity of CHD-APAH patients with reported pathogenic PAH gene variants might be due to the heterogeneous patient population with different complexity and severity of heart defects and/or the multifactorial pathogenesis of PAH.

The objective of this study was therefore to assess, whether pathogenic *SMAD6* variants can also be identified in CHD patients with PAH and in a second cohort of idiopathic PAH (IPAH) patients. Furthermore, we aimed to characterize the clinical phenotype of the patients with *SMAD6* variants.

## Results

**Fig. 1:**
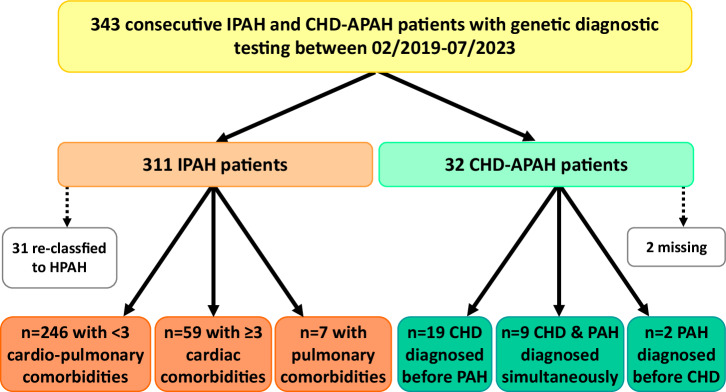
Flow diagram of the two study cohorts. Of the 343 included patients, 32 were diagnosed with congenital heart disease associated pulmonary arterial hypertension (CHD-APAH) and 311 with idiopathic PAH (IPAH). Of the latter 65 had cardiopulmonary comorbidities and in total 31 were re-classified to heritable PAH (HPAH). The majority of congenital heart defects was discovered prior to PAH diagnosis.

In this study we included 343 patients with PAH, 311 with IPAH and 32 with CHD-APAH (Table 1). CHD-APAH patients were diagnosed at an earlier age than IPAH patients (38 ± 21 years vs. 54 ± 18 years) and a greater proportion was female (81% vs. 68%). Out of the 311 patients with IPAH 65 were classified as IPAH with cardiopulmonary comorbidities (Fig. 1).

**Table 1:** Clinical patients’ characteristics

	CHD-APAH		IPAH patients	
Characteristics	Mean ± SD or %	n	Mean ± SD or %	n
Sex	81% female 19% male	32	68% female 32% male	311
Age at diagnosis (years)	38 ± 21	28	54 ± 18	303
6MWD (m)	413 ± 116	26	367 ± 130	265
NT-proBNP (ng/l)	496 ± 491	27	2056 ± 5886	271
WHO functional class	7% class 1	27	8% class 1	262
	37% class 2		42% class 2	
	44% class 3		44% class 3	
	11% class 4		6% class 4	
Current treatment	17% mono	29	33% mono	298
	48% double		39% double	
	31% triple		23% triple	
	3% quadruple		4% quadruple	
Hemodynamics at diagnosis				
mPAP (mmHg)	51.2 ± 18.3	28	47.0 ± 13.4	302
PAWP (mmHg)	10.3 ± 5.0	27	9.9 ± 3.8	293
PVR (WU)	8.0 ± 4.9	23	9.8 ± 5.3	280
Cardiac index (l/min/m²)	3.0 ± 0.9	23	2.3 ± 0.6	278
Cardiac output (l/min) (Thermodilution)	5.4 ± 2.0	23	4.2 ± 1.2	278
Cardiac output (l/min) (Fick)	5.9 ± 3.3	17	—	—

*6MWD* 6-minute walking distance, *CHD-APAH* congenital heart disease associated pulmonary arterial hypertension, *IPAH* idiopathic pulmonary arterial hypertension, *mPAP* mean pulmonary arterial pressure, *NT-proBNP* N-terminal pro-brain natriuretic peptide, *PAWP* pulmonary arterial wedge pressure, *PVR* pulmonary vascular resistance, *SD* standard deviation.

From the cohort with CHD-APAH 18 patients had heart defects of moderate or great complexity including two patients with a single ventricle, one with a double outlet right ventricle, one with transposition of great arteries. Four patients presented with an anomalous pulmonary venous return, one with an agenesis and one with an atresia of the right pulmonary artery and others with supravalvar aortic stenosis, VSD or ASD II with Eisenmenger syndrome or bicuspid aortic valve with an anomalous aortic origin of a coronary artery. Fourteen further patients presented with simple, isolated heart defects including small or closed VSD, ASD and PDA (Table 2). In the cohort of IPAH patients only a single, elderly patient had a documented aortic valve stenosis and a second patient a patent foramen ovale closure following a transient ischemic attack. No other heart defects were described.

**Table 2:** Types of congenital heart defects in the CHD-APAH cohort

Type of congenital heart defect	n
**Simple:**	
ASD (left to right shunt)	3
ASD closed	5
PDA open	1
PDA closed and persisting	1
VSD closed	4
**Moderate or great or complexity:**	
VSD with Eisenmenger	4
ASD with Eisenmenger	1
Combined aortic valve defect and supravalvar aortic stenosis	1
Transposition of great arteries	1
Single ventricle	2
Double outlet right ventricle with VSD	1
Anomalous pulmonary venous connection	5
Anomalous aortic origin of coronary artery and bicuspid aortic valve	1
Agenesis of right pulmonary artery	1
Atresia of right pulmonary artery	1

*ASD* atrial septal defect, *CHD-APAH* congenital heart disease associated pulmonary arterial hypertension, *PDA* patent ductus arteriosus, ventricular septal defect

Overall, we could identify likely pathogenic or rare missense *SMAD6* variants in six of the 343 patients (Tables 3–4), of which four (12.5%) were detected in the CHD-APAH cohort, and two (0.64%) in the IPAH cohort.

**Table 3:** Clinical characteristics of patients with rare *SMAD6* variants

Characteristics	Patient 1	Patient 2	Patient 3	Patient 4	Patient 5	Patient 6
Diagnosis	CHD-APAH	CHD-APAH	CHD-APAH	CHD-APAH	IPAH	IPAH with comorbidities
Congenital heart defects	Agenesis of right pulmonary artery, bicuspid aortic valve, ascending aortic aneurysm	Supravalvular aortic stenosis, combined pulmonary valve defect	ASD type 2, Eisenmenger syndrome	VSD surgically closed, atresia of the right pulmonary artery	none	none
CHD category	Great complexity	Moderate complexity	Moderate complexity	Great complexity	-	-
CHD presentation	Combined	Combined	Isolated	Combined	-	-
Variant(s) in *SMAD6*	frameshift	frameshift + missense	missense	missense	missense	frameshift
Inheritance	unknown	paternal + maternal	unknown	unknown	unknown	unknown
Sex	f	m	f	m	m	f
Age at diagnosis (years)	50	7 months	41	37	27	58
6MWD (m)	410	NA	NA	438	NA	345
NT-proBNP (ng/l)	224	3959	107	24	84	2256
WHO functional class	3	3	3	2	3	2
Current treatment	ERA + sGC stimulator + PCA	ERA + PDE5 inhibitor	ERA + PDE5 inhibitor	PDE5 inhibitor	PDE5 inhibitor	PDE5 inhibitor
Hemodynamics at diagnosis						
mPAP (mmHg)	29	65	40	37	34	32
PAWP (mmHg)	10	13	4	14	13	7
PVR (WU)	7.1	20 (PVRI)	7	3	3.09	8.25
Cardiac index (l/min/m²)	1.5	2.6	2.97	3.1	3.68	1.9
Cardiac output (l/min)	2.7	NA	5.14	7.7	6.8	3.1

*6MWD* 6-minute walking distance, *ASD* atrial septal defect, *CHD(-APAH)* congenital heart disease (associated pulmonary arterial hypertension), *ERA* endothelin receptor antagonist, *IPAH* idiopathic pulmonary arterial hypertension, *mPAP* mean pulmonary arterial pressure, *NT-proBNP* N-terminal pro-brain natriuretic peptide, *PAWP* pulmonary arterial wedge pressure, *PCA* prostacyclin analogue, *PDE5* phosphodiesterase 5, *PVR* pulmonary vascular resistance, *PVRI* pulmonary vascular resistance index, *sGC* soluble guanylate cyclase, *VSD* ventricular septal defect.

**Table 4:** *SMAD6* variants’ characteristics

Patient	Exon	DNA	Protein	Variant class (ACMG)^a^	CADD	REVEL	gnomAD v.2.1.1 controls allele frequency	gnomAD v.4.1.1 allele frequency	ClinVar / Literature
#1 CHD-APAH	1	c.592dupC	p.(Arg198Profs*105)	Likely pathogenic (PVS1 + 8, PM2_sup +1)	NA	NA	absent	absent	—
#2 CHD-APAH 1^st^ variant	1	c.590_602del	p.(Ser197Cysfs*41)	Likely pathogenic (PVS1 + 8, PM2_sup +1)	NA	NA	absent	absent	—
#2 CHD-APAH 2^nd^ variant	1	c.787C>T	p.(Pro263Ser)	VUS (PM1 + 2, PM2_sup +1, PP3 + 1)	26.9	0.92	absent	0.0003% Subpop._max_: 0.02%	4 x VUS^b^: 1 aortic valve disease, 1 complex CHD, 1 craniosynostosis, 1 inborn genetic disease
#3 CHD-APAH	2	c.851C>T	p.(Pro284Leu)	VUS	23.9	0.62	0.003% Subpop._max_: 0.03%	0.004% Subpop._max_: 0.006%	2 x VUS: 1 aortic valve disease, 1 inborn genetic disease
#4 CHD-APAH	4	c.1297G>A	p.(Gly433Ser)	VUS (PM1 + 2, PP3 + 1)	30	0.9	0.006% Subpop._max_: 0.04%	0.005% Subpop._max_: 0.006%	2 x VUS: 1 aortic valve disease; 1 aortic dissection^24^
#5 IPAH	1	c.731T>C	p.(Leu244Pro)	Likely pathogenic (PM1 + 2, PM2_sup +1, PP3 + 1, PM5 + 2)	28.8	0.77	absent	0.0006% Subpop._max_: 0.005%	1 x VUS: 1 aortic valve disease, congenital total pulmonary venous return anomaly
#6 PAH comorb.	1	c.107_128dup	p.(Glu44Trpfs*8)	Likely pathogenic (PVS1 + 8, PM2_sup +1)	NA	NA	absent	absent	1 x VUS: 1 aortic valve disease

^a^Variants were assessed considering ACMG criteria: American College of Medical Genetics, sore -1 to 5: VUS, 6–9: likely pathogenic; CADD: Combined Annotation Dependent Depletion tool, CADD scores ≥ 20: deleterious effect of variant assumed; CHD-APAH: pulmonary arterial hypertension associated with congenital heart disease; ClinVar: public database of genetic variants and their characterization; comorb.: comorbidities; gnomAD: genome Aggregation Database v2.1.1 with >60,000 healthy controls; IPAH: idiopathic pulmonary arterial hypertension; NA: not applicable; PM: pathogenic criterion moderate ( + 2); PP: pathogenic criterion supporting ( + 1), PVS: pathogenic criterion very strong ( + 8), REVEL: Rare Exome Variant Ensemble Learner, REVEL ≥ 0.75 deleterious missense effect assumed; _sup: downgraded to supporting criterion ( + 1); Subpop._max_: highest allele frequency in gnomAD v2.1.1 controls with at least 1000 alleles; VUS: variant of uncertain significance; *SMAD6* MANE transcript: NM_005585.5.

^b^The variant is listed 3x in ClinVar including 1 unknown phenotype submitted by GeneDX, representing 2 individuals.

### Genetic and clinical findings in CHD-APAH patients and family members

**Fig. 2:**
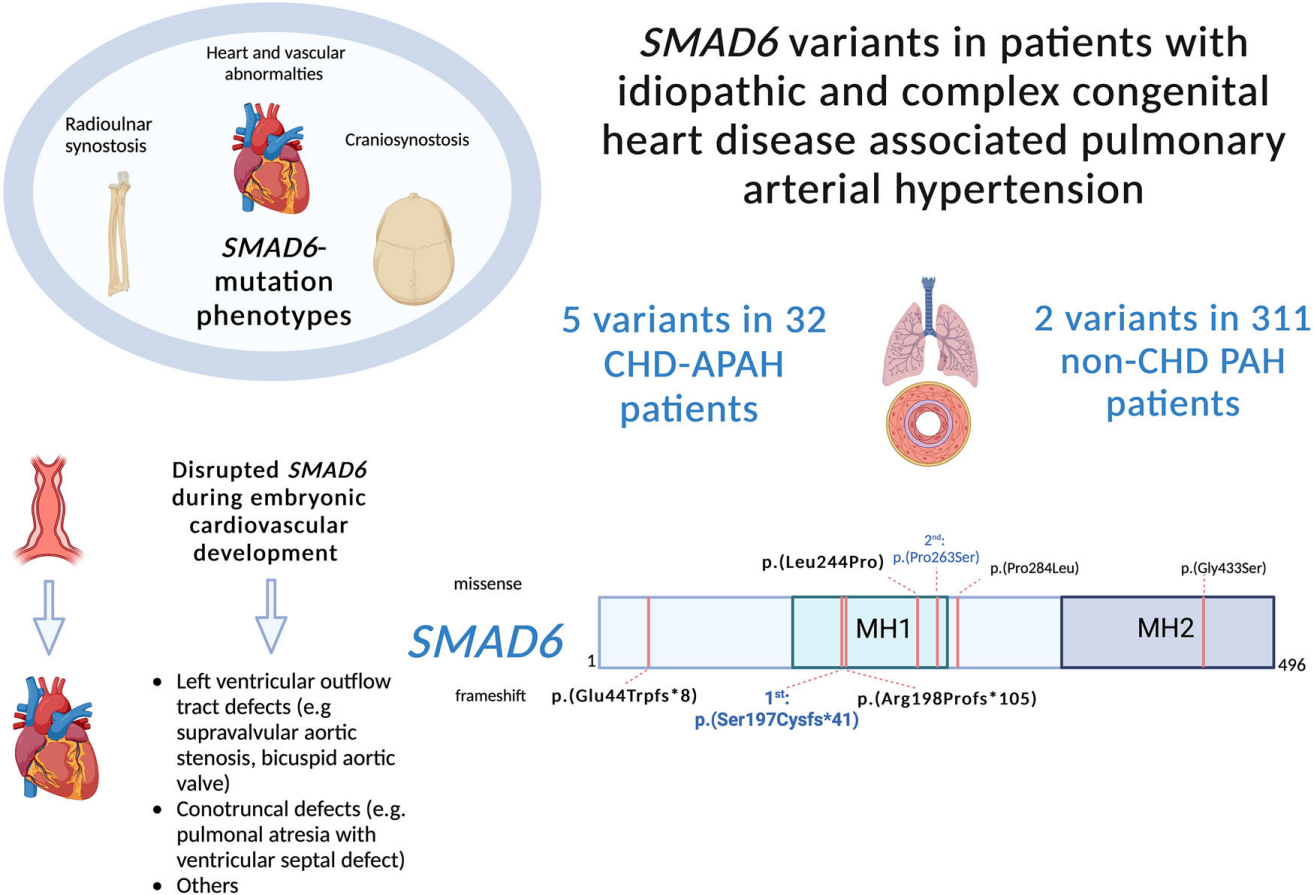
Central illustration with described phenotypes and roles for SMAD6 and *SMAD6* variants identified in this study. Pathogenic *SMAD6* variants have been previously described to cause radioulnar synostosis, heart defects and craniosynostosis. Heart defects result from disrupted embryonic cardiovascular development. This study identified 4 missense and 3 frameshift variants in 4 congenital heart disease associated pulmonary arterial hypertension (CHD-APAH) and 2 idiopathic PAH patients. Bold variants were classified as likely pathogenic. Created with BioRender.com.

In the cohort of 32 CHD-APAH patients we identified four patients with in total five rare potentially disease-causing *SMAD6* variants. The locations of the variants within the *SMAD6* gene are presented in Fig. 2. The first patient was diagnosed with PAH at the age of 50 years. At the same time an agenesis of the right pulmonary artery was detected and confirmed by cardiac MRI and pulmonary angiography. Additionally, the patient exhibited a bicuspid aortic valve and thoracic aortic aneurysm, both frequently identified in *SMAD6* pathogenic variant carriers^13^ (Table 3). The family history of the patient was negative for CHD or PAH. She had no children and her mother suffered from multiple sclerosis. Genetically we could identify a heterozygous frameshift variant in the *SMAD6* gene. The variant c.592dupC p.(Arg198Profs*105) was located in exon 1 of 4 and introduced a premature stop codon (Table 4). The variant was absent in more than 60,000 healthy controls (gnomAD v.2.1.1, also absent in gnomAD 4.1.0) and could be classified as likely pathogenic according to ACMG criteria.

The second patient was initially diagnosed with PAH in his first year of life with a complex heart defect consisting of a supravalvular aortic stenosis, stenosis of the left anterior descending artery and a combined pulmonary valve defect. Subsequently, the patient underwent commissurotomy of the aortic valve, enlargement plasty of the ascending aorta and left coronary sinus with an autologous pericardial patch. In further course, he underwent aortic valve replacement, aortoventriculoplasty, and reconstruction of the left coronary artery using a vena azygos interposition graft. Furthermore, he exhibited a short-segment Stanford A aortic dissection with intramural hematoma. Apart from his cardiac malformations and PAH, he also presented with bilateral radioulnar synostosis, craniosynostosis and dysmorphic facial features. At time of the clinical and genetic assessment within this study he was 28 years of age and clinically stable under targeted, double combination PAH therapy. This patient carried two genetic defects in *SMAD6*: the novel and likely pathogenic 13 base pair deletion c.590_602del p.(Ser197Cysfs*41) causing a frameshift and premature chain termination (Table 4). Furthermore, he harbored the additional missense variant c.787C>T p.(Pro263Ser) potentially contributing to the severity of his phenotype (Table 4). The missense variant was classified as deleterious by in silico prediction programs with a REVEL score of 0.92. It was already listed in the databank ClinVar for four patients, one of whom presented with an aortic valve disease, one with a complex CHD, one with craniosynostosis and one without submitted clinical details. In none of the patients listed in the databank ClinVar PAH has been described.

**Fig. 3:**
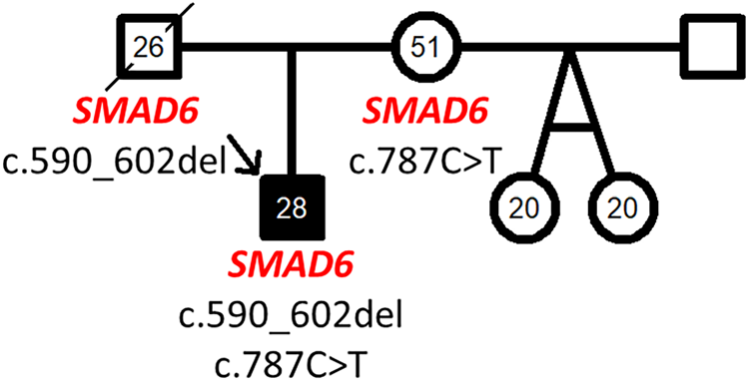
Pedigree of the second patient’s family with 2 *SMAD6* variants. The index patient was diagnosed at the age of 7 months and is now aged 28 years. He carried two biallelic *SMAD6* variants c.590_602del p.(Ser197Cysfs*41) and c.787C>T p.(Pro263Ser). Each variant was inherited from one parent. Circles denote females, squares males, strike through deceased individuals and filled symbols the presence pulmonary arterial hypertension.

When this index patient was 3 years old we had performed familial assessment using ECG and echocardiography at rest and during exercise and obtained blood for genetic analysis from both parents. His father also carried his *SMAD6* frameshift variant. He had an echocardiographic report at the age of 24 years which revealed a systolic pulmonary artery pressure of 45 mmHg at rest and an increased systolic pulmonary artery pressure during exercise with 87 mmHg at 150 watts. Additionally, a p-pulmonale was observed in the exercise ECG. The father had passed away two years after the assessment due to an accident without further assessment of his pulmonary hemodynamics. The missense variant of patient 2 was carried by his healthy mother (Fig. 3) confirming the bi-allelic nature of both variants in the index patient. Neither of the parents displayed any *SMAD6* associated phenotypes.

The third patient presented with CHD-APAH with an atrial septal defect (ASD) type 2 and currently resides in Eisenmenger’s syndrome stage without interventional or surgical closure options. She had three healthy children, while a pulmonary embolism was detected in her mother. The patient carried the missense variant in *SMAD6* c.851C>T p.(Pro284Leu), which had a total allele frequency of 0.003% and was detected in three healthy controls (gnomAD v.2.1.1 and in 72 individuals with unclear health status gnomAD v.4.1.0). Computational evidence from prediction programs was inconclusive (REVEL score 0.62), but this variant has been reported twice in ClinVar: once in a patient with aortic valve disease and once in a patient with an unspecified inborn genetic disease.

The fourth patient was diagnosed with CHD-APAH with atresia of the right pulmonary artery and a ventricular septal defect, which had been surgically corrected at the age of four years. He had no children, two sisters without any *SMAD6* characteristic phenotypes and his father succumbed to a cardiac infarction. He carried a missense variant, c.1297G>A p.(Gly433Ser), which had a total population allele frequency of 0.006%. In silico prediction programs classified this variant as deleterious (REVEL score 0.90). It was located in the highly conserved MH2 protein-binding domain of the SMAD6 protein. The same variant has been previously reported in a patient with an aortic valve disease and another one with an acute aortic dissection^19^.

Due to insufficient evidence at present, this missense variant and the two others were classified as variants of uncertain significance. In contrast, the frameshift variants of patient 1 and 2 most likely resulted in haploinsufficiency due to nonsense-medicated decay of the mRNA and were classified as likely pathogenic. No consanguinity was recorded in the families of the index patients.

It is important to note that loss-of-function (null) variants and missense variants are commonly found in the gene *SMAD6* in the gnomAD database. This leads to a low loss intolerance probability (pLI) score of 0 for null variants and a low missense Z-score of -7.6 representing a high tolerance in the general population to potentially deleterious *SMAD6* variants. We could, however, confirm a significant overrepresentation of *SMAD6* null (loss-of-function) variants in CHD-APAH patients (2 in 32 CHD-APAH patients vs. 830 in 807,162 gnomAD v.4.1.0 individuals; *p* = 0.0005). Moreover, a trend towards significant overrepresentation could be observed for missense variants in our CHD-APAH cohort (3 in 32 CHD-APAH patients vs. 21.680 in 807,162 gnomAD v.4.1.0 individuals; *p* = 0.054).

The patient characteristics of the CHD-APAH patients are summarized in Tables 1–2. Only one of the 28 CHD-APAH patients without a variant in *SMAD6* had another pathogenic PAH gene variant. It was the likely pathogenic heterozygous nonsense variant c.787C>T p.(Arg263*) in the gene *SMAD9*. No pathogenic variants in the *SOX17* or *TBX4* gene, any other established PAH gene nor sequenced candidate gene were identified in our CHD-APAH cohort.

### *SMAD6* variants in idiopathic PAH patients with and without comorbidities

Subsequently we extended our investigation to 246 IPAH patients without and 65 with cardiopulmonary comorbidities who received genetic diagnostic PAH-gene panel testing within the same time frame to screen for *SMAD6* variants of interest (Table 1).

Two further *SMAD6* variants were identified in one IPAH patient with and one without comorbidities, respectively (Tables 3–4, Fig. 2). The first IPAH patient was diagnosed at 27 years, had a negative family and no children. He carried the missense variant c.731T>C p.(Leu244Pro) classified as damaging by prediction programs (REVEL score 0.77). The missense variant was located in the highly conserved and functionally crucial MH1 DNA-binding domain^20^. The variant has been previously reported in a patient with a congenital total pulmonary venous return anomaly in ClinVar. Moreover, another missense variant at the same amino acid position (p.Leu244Arg) was identified in a patient with craniosynostosis^14^. It was experimentally characterized and led to significantly reduced protein stability^14^. With this additional evidence the variant could be classified as likely pathogenic.

The patient with IPAH and comorbidities suffered from chronic atrial fibrillation, diastolic left ventricular dysfunction and renal insufficiency. She had no children while her father had died of suspected right heart insufficiency. We detected the likely pathogenic frameshift variant c.107_128dup p.(Glu44Trpfs*8) in the *SMAD6* gene’s first exon. The variant was absent in population databases (gnomAD v2.1.1 and v4.1.0) and has been reported once in ClinVar in a patient with an aortic valve disease. Anamnestically, a history of aortic valve endocarditis, an antecedent aneurysm and dissection in the right groin, were noted. Additionally, the patient harbored the likely pathogenic, heterozygous missense variant in the *ACVRL1* gene c.775T>G p.(Phe259Val). None of the other *SMAD6* patients had a (likely) pathogenic variant in any of the PAH diagnostic genes. Of the other 309 IPAH patients 31 (10%) carried a disease-causing variant in one of the other PAH genes and could therefore be genetically re-classified as heritable PAH patients.

## Discussion

In this study we describe likely pathogenic or rare missense variants in the BMPR2 pathway gene *SMAD6* in 4 of 32 (12.5%) patients with CHD-APAH and in 2 out of 311 (0.64%) patients with IPAH. All of the former patients presented with complex CHD. Four of these in total seven variants were classified as likely pathogenic and could explain at least in part the patients’ phenotype, while three further variants were classified as variants of uncertain significance due to the lack of functional data clarifying the variant’s impact on the SMAD6 protein. One patient had bi-allelic *SMAD6* variants. In this patient we were able to identify for the first time all three so far known *SMAD6* associated phenotypes in a single individual including radioulnar synostosis, congenital heart defects and craniosynostosis in addition to PAH as a novel *SMAD6* phenotype.

In our cohort the sex distribution of *SMAD6* variant carriers was equal, even though 81% of the analyzed CHD-APAH and 68% of the IPAH patients were female, in line with previous reports^21^. In contrast, a male predominance has been observed among rare, deleterious *SMAD6* variant carriers with CHD (17:9)^13,14,22–25^. Both phenotypes occurring together may, therefore, offer an explanation for the equal sex-distribution of *SMAD6* variant carriers in our cohort.

All four CHD-APAH patients with *SMAD6* variants had a complex form of heart disease. A common finding for these rather different types of CHD is that the presence of PAH is not obligatory, even if the heart defects are left surgically untreated. It is known since long from a clinical standpoint that only part of these patients is prone to develop PAH, and screening for signs of CHD-APAH has always been prominently implicated in their follow-up. It is unclear why only a subset of patients with the same heart defect goes on to develop PAH. A potential trigger could be a genetic predisposition. To this end, our findings suggest that *SMAD6* may be a novel gene associated with CHD-APAH and potentially IPAH.

Previously reported cardiac malformations in *SMAD6* variant carriers were either complex cardiac defects and/or characterized by an aortic phenotype^13,14,22,24,25^. In all three *SMAD6* loss-of-function variant carriers in our study an aortic phenotype such as bicuspid aortic valve, aortic endocarditis or aortic stenosis has been identified. Such phenotypes were reported in 10/58 (17%) *SMAD6* loss-of-function variant carriers without PAH (Supplementary Table 1). While cardiac malformations have been previously described due to heterozygous pathogenic variants in the *SMAD6* gene^10–12^, only a single patient with PAH has been reported up to date^14^. In addition, it remains unclear why some *SMAD6* patients develop craniosynostoses^14^ and/or radioulnar synostoses^15^ and others a cardiac phenotype.

Only our bi-allelic *SMAD6* CHD-APAH patient, who was diagnosed at 7 months of age showed all of the other *SMAD6* phenotypes. In contrast, the remaining patients in our CHD-APAH cohort were diagnosed solely with congenital heart defects and PAH manifesting at a later stage in life. So far, 4 patients with bi-allelic variants in the *SMAD6* gene have been reported, exhibiting either more complex cardiac malformations, or featuring two of the three associated *SMAD6* phenotypes^22,23^.

The other *SMAD6* associated disorders are, just as PAH, inherited autosomal dominantly with a reduced penetrance^10^. This reduced penetrance was particularly evident in the family of the bi-allelic patient. The father of the index patient also carried the same likely pathogenic frameshift variant, yet did not exhibit congenital heart defects nor develop PAH. However, a slightly elevated systolic PAP during exercise echocardiography could be observed. Moreover, as he passed away at a young age due to an accident. PAH may still have manifested later in his life and it is inconclusive whether he had a higher vulnerability to cardiac conditions.

Retrospectively, the patient with IPAH and comorbidities and a likely pathogenic *SMAD6* frameshift variant displayed a susceptibility to cardiac and vascular events with an aortic valve endocarditis, aneurysm and dissection in the right groin. Interestingly, not only this but also four other *SMAD6* variants described in this study have been reported previously in patients with aortic valve disease albeit without PAH (ClinVar database, Table 4).

SMAD6, like SMAD7, is an inhibitory SMAD protein of the TGF-β superfamily. It predominantly targets the BMPR2 signaling pathway^26,27^. In the presence of abundant growth factors, SMAD6 is increasingly synthesized and acts as a negative feedback molecule^28^.

SMAD6 exhibits its inhibitory properties by several mechanisms. Firstly, it can bind to the BMP type 1 receptor, thereby preventing the phosphorylation of the receptor-related SMADs and inhibiting BMPR2 downstream signaling^26^. Secondly, SMAD6 recruits the ubiquitin ligase Smurf1 to the BMP type 1 receptor and facilitates receptor ubiquitination and subsequent degradation^29^. Thirdly, SMAD6 acts via non-canonical pathways such as the Notch signaling pathway^30^. SMAD6 is downstream of the mechanosensitive Notch 1 pathway, responsible for the flow-mediated cell alignment and barrier function of endothelial cells^30^. It is hypothesized that vascular remodeling in PAH is attributed, among other factors, to compromised endothelial barrier integrity and dysregulated endothelial proliferation^31^. The *SMAD6* variants could have therefore altered endothelial cell homeostasis and function.

Both, the TGF-β and the Notch signaling pathway, are involved in endothelial-to-mesenchymal transition (EndMT)^32^. Silencing of SMAD6 favors the development of a mesenchymal phenotype via the unimpaired BMP signaling pathway^33^. During EndMT, endothelial cells undergo a phenotypic shift, losing their characteristic features and acquiring mesenchymal traits. This process is believed to be implicated in various pathological conditions, including pulmonary hypertension^34,35^. Thus, a disturbed SMAD6 protein may drive mesenchymal cell features of the endothelium.

During embryonal development SMAD6 plays a key role in the endocardial cushion transformation^36^. Homozygous *Smad6* knockout mice exhibited higher lethality and various cardiac malformations, including valve hyperplasia and septation defects^37^. Surviving animals presented with aortic ossification, reduced vasodilation, and systemic hypertension. There was overexpression of mesenchymal cells in the heart valves, which could have resulted from an enhanced endocardial to mesenchymal transformation or increased cell proliferation according to Galvin and colleagues^37^.

Another potential mechanism of PAH development in *SMAD6* patients could be the presence of the underlying cardiac defects themselves. Two of our patients with *SMAD6* variants exhibited a rare segmental PAH characterized by an abnormal development of pulmonary vasculature due to atresia or agenesis of the pulmonary arteries. None of the patients with *SMAD6* variants had a simple heart defect as mainly reported for patients with pathogenic *SOX17* or *TBX4* variants. In contrast, all heart defects were of moderate to great complexity according to American Heart Association and American College of Cardiology guidelines^38^. Thus, the heart defect itself could have been the primary cause of PAH. However, not all patients with this phenotype develop PAH. Pathogenic *SMAD6* variants could, therefore, be an additional trigger for PAH manifestation offsetting the balance of the TGF-β pathway and the tightly controlled system of tissue homeostasis governed by proliferation and apoptosis.

While a great tolerance to frameshift variants in *SMAD6* exists in the general population (pLI score of 0), loss-of-function variants were significantly enriched in our CHD-APAH cohort and missense variants showed a trend of higher prevalence in our CHD-APAH cohort compared to the general population. An overrepresentation of rare missense and protein truncating *SMAD6* variants was also reported for CHD patients without PAH, in particular in those with bicuspid aortic valve and thoracic aortic aneurysms^10^. The elevated frequency of loss-of-function variants also in controls, may have obscured the detection of *SMAD6* as a potential novel CHD-APAH gene with reduced penetrance in former case-control studies.

Detailed clinical data and DNA samples from all index patients’ first-degree family members would have been very useful to perform further co-segregation analysis. Apart from the family of patient 2 no further DNA samples were available. From the recorded clinical family data, no further family members showed any signs of PAH or *SMAD6* associated phenotypes. Nevertheless, the presence of the *SMAD6* variants cannot be ruled out also within families 1 and 3–6 due to the reduced penetrance.

The prevalence of likely pathogenic *SMAD6* variants in our CHD-APAH patient cohort (6.3%) was higher than the reported frequency for *SOX17* (3.2%) or *TBX4* (2.6%) in CHD-APAH patients^16^. In contrast, *SMAD6* variants were not overrepresented in IPAH patients with or without comorbidities. Thus, it is unclear whether *SMAD6* variants also contributed to PAH development in the cohort of IPAH patients. Their classification as likely pathogenic does, however, offer at least some support for their disease contribution.

In summary, this is the first study to report *SMAD6* as a potentially novel IPAH and CHD-APAH gene. Further studies are necessary to confirm our findings and to determine the exact prevalence of disease-associated *SMAD6* variants. We advocate for testing *SMAD6* variants in PAH patients, particularly those with complex congenital heart defects or unclear cardiovascular abnormalities, as well as PAH patients with craniosynostosis or radial synostosis. Implementing *SMAD6* onto an existing PAH gene panel provides a feasible approach to routinely and cost-effectively screen for pathogenic *SMAD6* variants in patients with (CHD-A)PAH. Moreover, testing for *SMAD6* variants may positively influence patient management by enabling early detection and treatment of complications such as aneurysms. Similarly, CHD patients with a confirmed (likely) pathogenic *SMAD6* variant should be clinically evaluated for PAH aiming for early diagnosis and guideline-adherent management.

## Methods

### Patient cohorts and clinical characterization

We clinically and genetically assessed two sets of patients with PAH, a cohort of patients diagnosed with CHD-APAH and a second cohort diagnosed with IPAH with and without comorbidities. In both cohorts, PAH was diagnosed in the respective centers using the diagnostic algorithm and right heart catheterization according to current guidelines^3^ prior to genetic testing. CHD was diagnosed by electrocardiography, chest x-ray, transthoracic echocardiography, and cardiac magnetic resonance imaging, where indicated, following the respective guidelines^39^. Patients were classified as idiopathic when they had no further family members affected by PAH and no associated condition causing PAH such as connective tissue disease or CHD. Negative gene panel results corroborated the IPAH diagnosis. Patients with congenital heart disease were classified following the guidelines of the American Heart Association and American College of Cardiology^38^.

All IPAH and CHD-APAH patients had a mean pulmonary arterial pressure (mPAP) > 20 mmHg, a pulmonary arterial wedge pressure (PAWP) ≤ 15 mmHg, and a pulmonary vascular resistance (PVR) > 2 Wood units. PVR was calculated as the difference between mPAP and PAWP, divided by cardiac output (CO)^3^. CO was evaluated using thermodilution for IPAH patients and when possible calculated using the Fick equation for CHD-APAH patients. Cardiac index (CI) was calculated by dividing CO by body surface area. Precapillary pulmonary hypertension was defined as a mPAP > 20 mmHg, PAWP ≤ 15 mmHg, and PVR > 2 Wood units^3^. In patients with CHD-APAH further assessments were performed to characterize the heart defects including cardiac magnetic resonance imaging (MRI), left heart catheterization, and pulmonary angiography as appropriate for correct diagnosis. Heart defects were classified by the adult congenital heart disease anatomic classification^38^. At diagnosis, World Health Organization (WHO) functional class was recorded, echocardiography, 6-minute walking test and laboratory parameters were measured including N-terminal pro-brain natriuretic peptide (NT-proBNP).

Cardiac comorbidities included systemic atrial hypertension, diabetes mellitus type 2, body mass index >30 kg/m^2^, coronary artery disease, left atrial enlargement and atrial fibrillation. Pulmonary comorbidities were defined by mild interstitial lung disease or mild chronic obstructive pulmonary disease.

Some of the underlying heart defects were initially diagnosed externally and patients were later evaluated for PAH at pulmonary hypertension expert centers. Patients received genetic counseling and provided written informed consent for the scientific use of their samples. Genetic testing was performed at the Institute of Human Genetics at Heidelberg University. The Ethics Committee at the Medical Faculty of Heidelberg University had no objections against this study (project identification code S-426/2017 and S-925/2020). The authors confirm that the study conforms to recognized standards of European Medicines Agency Guidelines for Good Clinical Practice and the Declaration of Helsinki for medical research involving human subjects.

### Genetic testing

EDTA blood samples from patients with the diagnosis of IPAH or CHD-APAH given by the treating PH expert were consecutively sent between February 2019 and July 2023 for genetic testing of PAH genes (Fig. 1). DNA was extracted using an automated procedure (Autopure or QIAsymphony, QIAGEN, Germany). Using our patented PAH specific gene panel (EP3507380) samples were sequenced based on next-generation sequencing (NGS) using a customized SureSelect QXT kit (Agilent, Germany) on the MiSeq (Illumina, USA) following the manufacturer’s instructions. PAH diagnostic genes were expanded over time to include the most recently identified and validated genes as described previously^8^ including the 18 diagnostic genes: *ABCC8* (HGNC:59), *ACVRL1* (HGNC:175)*, BMPR1B* (HGNC:1077)*, BMPR2* (HGNC:1078)*, CAV1* (HGNC:1527)*, EIF2AK4* (HGNC:19687)*, ENG* (HGNC:3349)*, GDF2* (HGNC:4217)*, KCNA5* (HGNC:6224)*, KCNK3* (HGNC:6278)*, KDR* (HGNC:6307)*, KLF2* (HGNC:6347)*, SMAD4* (HGNC:6770)*, SMAD9* (HGNC:6774), *TBX4* (HGNC:11603), *ATP13A3* (HGNC:24113)*, AQP1* (HGNC:633), and *SOX17* (HGNC:18122). All coding regions as well as exon-intron boundaries were analyzed including 20 base pair up- and downstream of each exon. Additionally, up to 46 research genes including the SMAD genes *SMAD1* (HGNC:6767)*, SMAD5* (HGNC:6771)*, SMAD6* (HGNC:6772)*, SMAD7* (HGNC:6773) were examined for all patients using the same procedure. For this study, only *SMAD6* variants were screened in addition to diagnostic genes in IPAH patients. Familial variants were investigated by Sanger sequencing (ABI Genetic Analyzer 3130xl, Applied Biosystems, USA) according to manufacturer’s instructions.

### Variant characterization

The quality of each NGS run was assessed based on the volume of generated data and an average base call error rate of ≤0.01% ( ≥Q30). Coverage was maintained at a minimum of 100 reads. Identified variants in the MANE transcript of each gene were compared to the human reference genome (GRCh37).

Allele frequencies were sought in the genome aggregation database (gnomAD v.4.1.0, 807.162 individuals with a mixed health background) as well as in the subset of more than 60.000 healthy controls in gnomAD v.2.1.1. Variants with an allele frequency exceeding 1% in the population were classified as benign and were filtered out. Missense variants were evaluated for their deleteriousness using the prediction programs CADD and REVEL. The variants were characterized based on a point-based system according to the American College of Medical Genetics and Genomics (ACMG) criteria and categorized into the classes benign (1), likely benign (2), variant of uncertain significance (3), likely pathogenic (4), and pathogenic (5)^40–42^. Subsequently, variants were queried in the databases HGMD and ClinVar, and a literature search was performed.

### Statistical analyses

Descriptive statistics were presented as mean with standard deviation or frequency and corresponding percentage. We utilized the exact Fisher´s t-test to statistically test for an enrichment of the number of *SMAD6* missense or loss-of-function variants in our CHD-APAH cohort compared to those variants described in gnomAD v.4.1.0 containing more than 800,000 individuals with an unknown health status from different ethnicities. Statistical significance was considered when *p* < 0.05.

## Supplementary information

Supplementary Information

## Data Availability

All data generated or analysed during this study are included in this published article.
