# Supplementary material for: Pathogenic SMAD6 variants in patients with idiopathic and complex congenital heart disease associated pulmonary arterial hypertension
Source: NPJ Genom Med. 2025 Mar 25;10:28. doi: 10.1038/s41525-025-00484-6 (PMC11937313; doi:10.1038/s41525-025-00484-6)
Supplement: Supplementary file 1 — Supplementary Information [file 41525_2025_484_MOESM1_ESM.pdf]

Supplementary Information

Supplementary table 1: Phenotypes of *SMAD6* loss-of-function patients (extended from [1])

DNA change	Protein change	Patient phenotype	Aortic (valve) disease?	Inheritance	Reference
c.42G>A	p.(Trp14*)	Bicuspid aortic valve/thoracic aortic aneurysm	yes	unknown	1
c.43C>T	p.(Arg15*)	Metopic craniosynostosis	no	transmitted	2
c.95del	p.(Gly32Alafs*32)	Metopic craniosynostosis, mild-moderate delay, mild dymorphism, small VSD and ASD, resolved by 2 years	no	not from mother	3
c.106dup	p.(Asp36Glyfs*9)	RUS: bilateral type I	no information	de novo	4
c.107_128dup	p.(Glu44Trpfs*8)	Idiopathic pulmonary arterial hypertension, aortic valve endocarditis, antecedent aneurysm, dissection of iliac artery; atrial fibrillation, diastolic left ventricular dysfunction, renal insufficiency	yes	unknown	This study Patient 6
c.124C>T	p.(Arg42*)	Metopic craniosynostosis; ventriculomegaly, absent corpus callosum	no	de novo	3
c.223C>T	p.(Arg75*)	RUS: right type I, macrocephaly	no information	unknown	4
c.232_250del	p.(Gln78Glyfs*41)	Sagittal and metopic craniosynostosis	no	de novo*	5
c.258_259dup	p.(Gly87Glufs*39)	Hypoplastic left heart syndrome, hypoplastic left ventricle, aortic arch hypoplasia, aortic atresia, hypoplasia ascending aorta, mitral atresia, restrictive PFO	yes	transmitted	6
c.263_264delinsT	p.(Gly88Valfs*37)	RUS: left type II,right type II, phalangeal fusion, macrocephaly	no information	maternal	4
c.264_265insG	p.(Pro89Alafs*32)	Metopic craniosynostosis	no	de novo	2
c.345G>A	p.(Trp115*)	RUS: left type I	no information	unknown	4
c.384_385del	p.(Ser130Glyfs*172)	Metopic craniosynostosis	no	transmitted	2
c.389C>A	p.(Ser130*)	RUS: bilateral type I, microcephaly	no information	unknown	4
c.408dup	p.(Ala137Glyfs*166)	PFO, pulmonary stenosis (subvalvar), tetralogy of Fallot, VSD (malalignment), coronary artery anomaly	no	de novo	6
c.433dup	p.(Leu145Profs*158)	Sagital and left coronal craniosynostosis; moderate-severe delay; delayed dental eruption; class II malocclusion	no	de novo	3
c.442del	p.(Ala148Argfs*33)	RUS: bilateral type I	no information	paternal	4
c.452_458del	p.(Glu151Glyfs*28)	RUS: bilateral type II	no information	maternal	4
c.455_461del	p.(Pro152Profs*27)	Bicuspid aortic valve/thoracic aortic aneurysm	yes	transmitted	7

DNA change	Protein change	Patient phenotype	Aortic (valve) disease?	Inheritance	Reference
c.455_461del	p.(Pro152Profs*27)	Sagittal craniosynostosis	no	transmitted	[2]
c.465_471dup	p.(Ser158Argfs*147)	Metopic craniosynostosis	no	paternal	[3]
c.465_471dup	p.(Ser158Argfs*147)	Metopic craniosynostosis, macrocephaly (+5.1 SD), mild prominence 3rd and lateral ventricles	no	unknown	[3]
c.465_471dup	p.(S158Rfs*147)	RUS: left type I	no information	unknown	[4]
c.465_471del	p.(G156Vfs*23)	RUS: bilateral type II	no information	maternal	[4]
c.465_471del	p.(G156Vfs*23)	RUS: left type I	no information	de novo	[4]
c.465_471del	p.(G156Vfs*23)	RUS: bilateral type I	no information	de novo	[4]
c.465_471del	p.(G156Vfs*23)	RUS: left type I	no information	unknown	[4]
c.577G>T	p.(Glu193*)	ASD (secundum), left-sided PDA, tubular hypoplasia of aorta, coarctation of aorta, VSD (malalignment, muscular outlet)	yes	transmitted	[6]
c.589del	p.(S197Pfs*45)	RUS: bilateral type I, overheight, macrocephaly	no information	de novo	[4]
c.590_602del	p.(Ser197Cysfs*41)	Pulmonary arterial hypertension, supravalvular aortic stenosis, combined pulmonary valve defect	yes	paternal	This study Patient 2
c.592dupC	p.(Arg198Profs*105)	Pulmonary arterial hypertension, Agenesis of right pulmonary artery, bicuspid aortic valve, ascending aortic aneurysm	yes	unknown	This study Patient 1
c.667C>T	p.(Gln223*)	Metopic craniosynostosis	no	transmitted	[5]
c.726del	p.(Lys242Asnfs*297)	Bicuspid aortic valve/thoracic aortic aneurysm	yes	unknown	[7]
c.775del	p.(Val259Cysfs*280)	Right coronal craniosynostosis, severe visual impairment (also in mother)	no	maternal	[3]
c.794del	p.(His265Profs*274)	Bicuspid aortic valve/thoracic aortic aneurysm	yes	transmitted	[1]
c.837C>A	p.(Tyr279*)	Bicuspid aortic valve/thoracic aortic aneurysm	yes	unknown	[7]
c.837C>G	p.(Tyr279)*	Metopic craniosynostosis	no	maternal	[3]
c.837C>G	p.(Tyr279*)	RUS: right type I	no information	maternal	[4]
c.[839C>T; 1041T>A]	p.(Ser280Phe; Tyr347*)	Sagital craniosynostosis	no	maternal	[3]
c.839dup	p.(Arg281Serfs*22)	Sagittal craniosynostosis	no	transmitted	[5]
c.864C>G	p.(Tyr288*)	Bicuspid aortic valve/thoracic aortic aneurysm	yes	unknown	[7]
c.900C>A	p.(Tyr300*)	RUS: bilateral type I, Pectus carinatum, macrocephaly	no information	unknown	[4]
c.932_933del	p.(Thr311Serfs*4)	Tetralogy of Fallot	no	transmitted	[6]
c.943G>T	p.(Glu315*)	RUS: bilateral type I	no information	unknown	[4]

DNA change	Protein change	Patient phenotype	Aortic (valve) disease?	Inheritance	Reference
c.1034del	p.(Arg345Pro*fs194)	Sagittal and metopic craniosynostosis	no	transmitted	5
c.1050C>G p.	p.(Tyr350*)	RUS: right type I	no information	paternal	4
c.1050C>G	p.(Tyr350*)	RUS: right type II	no information	unknown	4
c.1055_1056insAT	p.(Ala353Trpfs*187)	Sagittal and metopic craniosynostosis	no	transmitted	5
c.1057_1058del	p.(Ala353Argfs*211)	Aortic arch hypoplasia, bicuspid aortic valve, coarctation of aorta, PFO, hypoplastic left ventricle	yes	transmitted	6
c.1074C>A	p.(Thr358*)	Aberrant left subclavian artery, abnormal branching right aortic arch, right aortic arch ligament, vascular ring	yes	transmitted	6
c.1102del	p.(Leu368Trpfs*171)	Metopic craniosynostosis, **mother of patient with pulmonary arterial hypertension**	no	**maternal**	3
c.1119_1123dup	p.(Gln375Argfs*166)	Metopic craniosynostosis	no	paternal	3
c.1120G>T	p.(Glu374*)	Sagittal craniosynostosis	no	de novo	5
c.1156A>T	p.(Lys386*)	Sinus venosus septal defect (superior type), partially anomalous pulmonary veins	no	transmitted	6
c.1219G>T	p.(Glu407*)	Metopic craniosynostosis	no	transmitted	5
c.1296dup	p.(Gly433Argfs*132)	Metopic craniosynostosis, small PFO resolved	no	paternal	3
c.1302C>G	p.(Tyr434*)	Transposition of great arteries	no	transmitted	6
c.1304_1313dup	p.(Phe439Hisfs*129)	RUS: bilateral type I	no information	maternal	4
c.1304_1313dup	p.(Phe439Hisfs*129)	RUS: left type I, right type II	no information	paternal	4
c.1339C>T	p.(Gln447*)	RUS: bilateral type I	no information	maternal	4
c.1419_1420del	p.(Pro474Leufs*90)	Pulmonary stenosis (subvalvar, valvar), tetralogy of Fallot	no	transmitted	6

ASD: atrial septal defect; PDA: patent ductus arteriosus; PFO: patent foramen ovale; RUS: radioulnar synostosis type I (proximal fusion of radius and ulnar) and/or type II: fusion distal to proximal radial epiphysis with dislocation of the radius head; VSD: ventricle septal defect

Supplementary information

The up to 46 research genes sequenced in addition to PAH-specific diagnostic genes included:

ACVR1, ANXA11, BMP2, BMP7, BMP10, BMPR1A, BTNL2, COX4I2, COX5A, CREB1, CYP1B1, EPAS1, FBLN2, FLNA, FOXF1, FOXO1, GGCX, GUCY1A3, HRG, ID1, ID2, ID3, ID4, IL6, JAK2, KLF4, KLF5, KLK1, NOTCH3, PDGFD, PHF14, RASA1, SMAD1, SMAD5, SMAD6, SMAD7, SMYD2, SOD2, TBX2, TET2, THBS1, TMEM70, TOPBP1, VCAN, VHL, ZFYVE16.

Supplementary references:

1 Luyckx, I. *et al.* Confirmation of the role of pathogenic SMAD6 variants in bicuspid aortic valve-related aortopathy. *Eur J Hum Genet* **27**, 1044-1053 (2019). https://doi.org/10.1038/s41431-019-0363-z

2 Timberlake, A. T. *et al.* De novo mutations in inhibitors of Wnt, BMP, and Ras/ERK signaling pathways in non-syndromic midline craniosynostosis. *Proc Natl Acad Sci U S A* **114**, E7341-E7347 (2017). https://doi.org/10.1073/pnas.1709255114

3 Calpena, E. *et al.* SMAD6 variants in craniosynostosis: genotype and phenotype evaluation. *Genet Med* **22**, 1498-1506 (2020). https://doi.org/10.1038/s41436-020-0817-2

4 Yang, Y. *et al.* SMAD6 is frequently mutated in nonsyndromic radioulnar synostosis. *Genet Med* **21**, 2577-2585 (2019). https://doi.org/10.1038/s41436-019-0552-8

5 Timberlake, A. T. *et al.* Two locus inheritance of non-syndromic midline craniosynostosis via rare SMAD6 and common BMP2 alleles. *Elife* **5** (2016). https://doi.org/10.7554/eLife.20125

6 Jin, S. C. *et al.* Contribution of rare inherited and de novo variants in 2,871 congenital heart disease probands. *Nat Genet* **49**, 1593-1601 (2017). https://doi.org/10.1038/ng.3970

7 Gillis, E. *et al.* Candidate Gene Resequencing in a Large Bicuspid Aortic Valve-Associated Thoracic Aortic Aneurysm Cohort: SMAD6 as an Important Contributor. *Front Physiol* **8**, 400 (2017). https://doi.org/10.3389/fphys.2017.00400
